# Age-Related Decline of Low-Spatial-Frequency Bias in Context-Dependent Visual Size Perception

**DOI:** 10.3389/fpsyg.2019.01768

**Published:** 2019-07-31

**Authors:** Anqi Wang, Shengnan Zhu, Lihong Chen, Wenbo Luo

**Affiliations:** Research Center of Brain and Cognitive Neuroscience, Liaoning Normal University, Dalian, China

**Keywords:** aging, spatial frequency, Ebbinghaus illusion, faces, awareness

## Abstract

Global precedence has been found to decline or even shift to local precedence with increasing age. Little is known about the consequence of this age-related decline of global precedence on other aspects of older adults’ vision. The global and local processing has been preferentially associated with the low-spatial-frequency (LSF) and high-spatial-frequency (HSF) channels, respectively. Here, we used low- and high-pass filtered faces together with the Ebbinghaus illusion whose magnitude is an index of context sensitivity. The results demonstrated that, relative to HSF faces, prior exposure to LSF faces increased the illusion magnitude for younger participants, but it reduced the illusion magnitude for older participants. Significant age group difference was observed only with prior exposure to LSF faces but not to HSF faces. Moreover, similar patterns of results were observed when the filtered faces were rendered invisible with backward masking, and the magnitude of age-related decline was comparable to the visible condition. Our study reveals that LSF-related enhancement of context sensitivity declines with advancing age, and this age-related decline was independent of the awareness of the spatial frequency information. Our findings support the right hemi-aging model and suggest that the magnocellular projections from subcortical to cortical regions might also be vulnerable to age-related changes.

## Introduction

Aging is associated with a decline in many aspects of visual processing, including global and local processing^1^, which may affect the everyday functioning and well-being of older adults. Converging studies have found that the global precedence effect [i.e., faster response times (RTs) to global forms relative to local forms of hierarchical stimuli^2^ and more interference from global forms to local forms] declines or even shifts to a local precedence effect with increasing age. For example, by using Navon stimuli (i.e., large letters shaped by small letters), Staudinger et al. (2011) found no significant difference of RTs between global and local targets for older adults, though younger adults exhibited a significant global RT advantage. Several studies, using a variety of hierarchical stimuli (Oken et al., 1999; Slavin et al., 2002; Lux et al., 2008; Lithfous et al., 2016), found that healthy older adults are quicker and more accurate to detect local targets than global targets. Meanwhile, imaging studies have found age-related decline of structural and functional architecture of human cortical brain, which is hemispherically asymmetric, with the decline being more predominant in the right than the left hemisphere (Resnick et al., 2003; Li et al., 2009; Lu et al., 2011; Jockwitz et al., 2017).

What consequence does the age-related decline of global precedence have on other aspects of older adults’ visual processing? It has been found that, for younger adults, global processing bias can enhance context sensitivity relative to local processing bias. For instance, Mundy (2014) found that when younger adults were prior exposed to global or local forms of Navon stimuli, the magnitude of the Müller-Lyer illusion which was measured subsequently was significantly increased for participants with a global processing bias and was significantly reduced for those with a local processing bias, in contrast to a control condition. People who have a tendency toward local processing demonstrate a smaller strength of the Ebbinghaus illusion comparing to their controls (de Fockert et al., 2007; Davidoff et al., 2008a,b). Similar results have been observed when using spatial frequency (SF) information and the Ebbinghaus illusion. Previous studies have suggested an intimate relationship between SF information and global/local processing (Shulman et al., 1986; Shulman and Wilson, 1987; Badcock et al., 1990; Hughes et al., 1990; Jiang and Han, 2005), both of which demonstrate hemispheric asymmetry (Fink et al., 1996; Peyrin et al., 2004) and are mapped retinotopically in the occipital cortex (Sasaki et al., 2001; Henriksson et al., 2008; Musel et al., 2013). It has been proposed that differences in the global and local processing may be related to underlying properties of channels involved in the LSF and HSF processing, respectively (Hughes et al., 1990; Boeschoten et al., 2005). Chen et al. (2018) found that when younger participants were prior exposed to LSF information, the magnitude of the Ebbinghaus illusion which was measured subsequently was significantly increased relative to prior exposure to HSF information. Therefore, we conjectured that the decline of global precedence with advancing age might reduce the context sensitivity. To test this hypothesis, we used the paradigm similar to Chen et al. (2018). Specifically, younger and older participants were prior exposed to low- or high-pass filtered fearful faces and then were presented with the Ebbinghaus configuration. We predicted that, for the younger participants, prior exposure to LSF faces would increase the illusion strength relative to HSF faces. However, this LSF-related enhancement of context sensitivity would decline or even reverse for the older participants.

Further, accumulating evidence suggests that the processing of the global forms of hierarchical stimuli can take place without awareness. For example, the global priming effect can be observed when the priming stimulus (i.e., a Navon stimulus) is rendered invisible with binocular suppression (Koivisto and Revonsuo, 2004). The global interference persists even when the Navon stimuli are presented for short durations of 10 ms (Hibi et al., 2002) and 17 ms (Andres and Fernandes, 2006). Moreover, an older patient who denied any awareness of the global forms of Navon stimuli due to substantial atrophy in posterior cortical regions showed a normal pattern of global interference when asked to identify local targets (Filoteo et al., 2002). Therefore, we predicted that the decline of LSF-related enhancement of context sensitivity associated with aging might be independent of the awareness of LSF information. To examine this possibility, we rendered the filtered faces invisible with backward masking. We expected that the age-related decline should be observed in this invisible condition, and the magnitude of the decline should be comparable to the visible condition.

## Materials and Methods

### Participants

A total of 45 participants took part in the study, with 20 participants in the younger group (8 male, mean age = 22.0 years, SD = 2.1, age range = 20–28) and 25 participants in the older group (10 male, mean age = 64.2 years, SD = 3.3, age range = 60–70). All participants had normal or corrected-to-normal eyesight and were screened using an array of visual and cognitive tests before the study. None reported visual pathology (e.g., glaucoma or cataracts) or neurological history. The study was approved by the institutional review board of Liaoning Normal University, and it adhered to the tenets of the Declaration of Helsinki. All participants provided informed consent and were naïve to the purpose of the experiments.

There was no significance between the two age groups with respect to gender distribution (*χ*^2^ = 0, *p* = 1.000), visual acuity [*t*(43) = −0.93, *p* > 0.250], and contrast sensitivity [*t*(43) = 1.57, *p* = 0.125]. Younger group had longer education years [*t*(43) = 5.60, *p* < 0.001] and higher Raven Standard Progressive Matrices (SPM) scores [*t*(43) = 4.29, *p* < 0.001] than older group (Table 1).

**Table 1:** Demographic information and results from visual and cognitive tests administered prior to the start of the study.

Variable	Younger		Older	
	*M*	SD	*M*	SD
Age (years)^*^	22.00	2.08	64.20	3.25
Education (years)^*^	14.85	1.27	11.32	2.58
Log contrast sensitivity	1.82	0.29	1.68	0.33
Visual acuity (LogMAR units)	−0.03	0.22	0.03	0.19
SPM score^*^	55.00	3.85	46.20	8.48

*Contrast sensitivity and visual acuity were measured using a computer-based Freiburg Vision Test at a distance of 250 cm (Bach, 1996). Participants’ intelligence was assessed by using the non-verbal Raven Standard Progressive Matrices (SPM, China Revised edition, 1989) (Zhang and Wang, 1989). LogMAR, logarithm of the minimum angle of resolution*.

* *For these tests, the differences between the two age groups were significant (ps < 0.001)*.

### Stimuli

Stimuli were displayed using MATLAB (The MathWorks, Natick, MA) together with the Psychophysics Toolbox extensions (Brainard, 1997; Pelli, 1997). The Ebbinghaus configuration was composed of a central circle (1.1° × 1.1°) surrounded by four large (1.7° × 1.7°) or small (0.6° × 0.6°) circles. The initial size of a comparative circle was varied from trial to trial ranging from 0.86° to 1.37° in 0.06° steps. Fearful face images (3.4° × 5.1°) with an equal number of male and female (2 male and 2 female) were selected from the NimStim set of facial expressions (Tottenham et al., 2009), with all hair and nonfacial features being removed. The face images were passed through a second-order Butterworth filter, using a high-pass cutoff (above 6 cpd) for HSF faces and a low-pass cutoff (below 2 cpd) for LSF faces, following previous studies (Schyns and Oliva, 1999; Chen et al., 2018). The filtered faces were assigned identical average luminance value (114 cd/m^2^) and root mean square contrast using the SHINE toolbox for MATLAB (Willenbockel et al., 2010). Participants were positioned 57 cm from a gray computer screen (gamma corrected, 128 cd/m^2^, 1,440 × 900 at 60 Hz) with their head stabilized in a chin rest.

### Procedure

**Figure 1:**
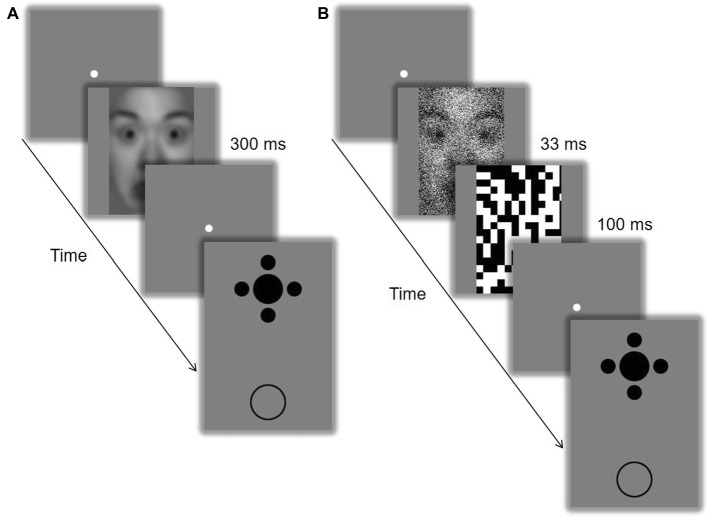
Schematic representation of the experimental procedures. In Experiment 1 **(A)**, the filtered face was presented for 300 ms, followed by the Ebbinghaus configuration. In Experiment 2 **(B)**, the filtered face was presented for 33 ms followed by a black-and-white random-noise mask. The identifiable image is model #18 from the NimStim Set of Facial Expressions (Tottenham et al., 2009) and is released for publication in scientific journals.

In Experiment 1, the procedure included two sessions (i.e., LSF and HSF), with an interval of at least 1 day. The session sequence was counterbalanced between participants. Each session was composed of two phases. During the pre-test phase, participants were repeatedly presented with a LSF or HSF face for 300 ms and had to discriminate the gender of the faces as accurately and quickly as possible with key presses. There was a total of 160 trials with 80 repetitions for each condition. During the test phase following immediately (Figure 1A), the filtered faces were presented for 300 ms at the beginning of each trial to strengthen the SF bias produced by the pre-test phase. Participants were asked to perform the gender discrimination task. They were then displayed with the Ebbinghaus configuration at the screen center and a comparative circle in the lower visual field. They had to adjust the size of the comparative circle with key presses to match that of the central target without time limit. There was a total of 160 trials with 40 repetitions for each condition (SF of stimuli: low vs. high; size of inducers: large vs. small). Participants received no feedback about their accuracy of gender discrimination during both the pre-test and the test phases.

In Experiment 2, the procedure and stimuli were similar to those of Experiment 1, except that a black-and-white random-noise mask was presented after the offset of the filtered face, which had noise on it and was displayed for a shorter duration of 33 ms (Figure 1B).

## Statistical Analysis

The perceived size of the central target was calculated as follows: $\frac{\mathrm{measured size}-\mathrm{physical size}}{\mathrm{physical size}}\;\;\times \;\;100.$ The measured size was the size of the adjusted comparative circle that match the central target, and the physical size was referred to as that of the central target (1.1°). The illusion magnitude was measured as the differences of the perceived sizes of the central targets surrounded by small and large inducers. For Experiment 1, RTs of gender discrimination and perceived sizes of the targets were collected from the trials with correct responses. For Experiment 2, all trials had been included for the analysis of the perceived sizes of the targets. The perceived size of the target circle was entered into a 2 × 2 × 2 mixed analysis of variance (ANOVA) with SF of stimuli (low vs. high) and size of inducers (large vs. small) as within-subject factors and age group (younger vs. older) as a between-subject factor. To draw definite conclusions about the viability of the null hypothesis, Bayes factor (BF) with Cauchy distribution (scale *r* = 1) was used to denote the likelihood of the alternative (H_1_) over the null (H_0_) hypothesis (Rouder et al., 2009).

## Results

### Experiment 1

**Figure 2:**
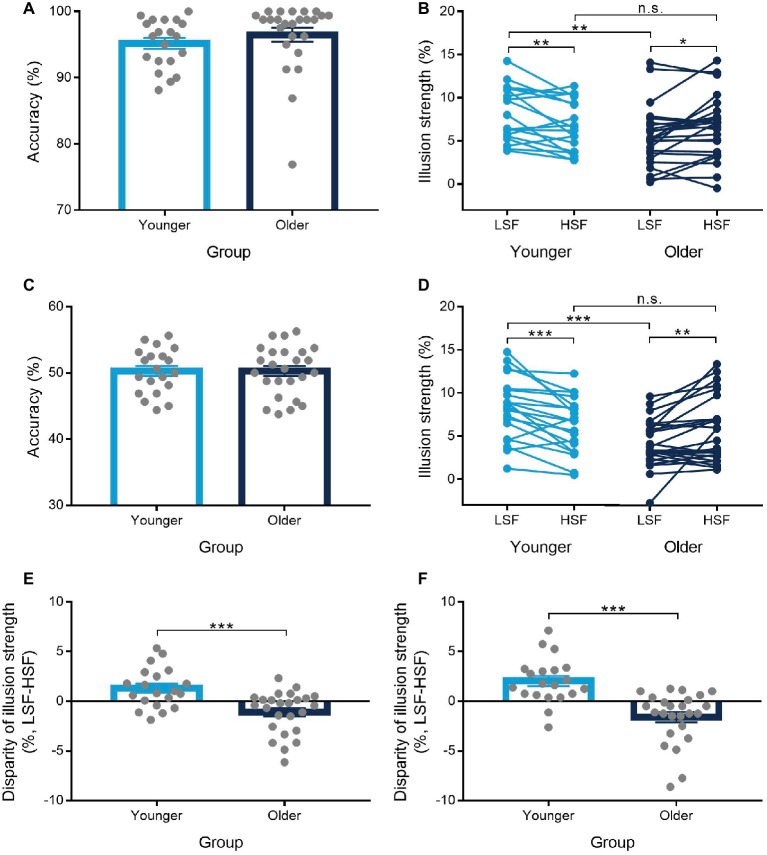
Results from Experiments 1 and 2. Accuracy of gender discrimination task during the test phase in Experiments 1 **(A)** and 2 **(C)**, the illusion strength as a function of low-spatial-frequency (LSF) and high-spatial-frequency (HSF) conditions in Experiments 1 **(B)** and 2 **(D)**, and the disparity of illusion strength between the LSF and HSF conditions in Experiments 1 **(E)** and 2 **(F)**. Error bars represent one standard error of the mean. Asterisks (*) indicate significance level of ^*^p < 0.05, ^**^p < 0.01, and ^***^p < 0.001.

The proportions of correct responses on gender discrimination task were above chance level in the two phases for both the younger (pre-test: *M* = 92.4%; test: *M* = 95.2%) and the older (pre-test: *M* = 93.7%; test: *M* = 96.5%; see Figure 2A) groups. The difference between the LSF and HSF conditions was not significant with accuracy and RTs during the pre-test and the test phases for the two age groups (Table 2). For the size matching task, results from ANOVA demonstrated a significant main effect of size of inducers [*F*(1,43) = 212.93, *p* < 0.001, $\eta_{p}^{2}$ = 0.83] and a significant main effect of SF of stimuli [*F*(1,43) = 5.25, *p* = 0.027, $\eta_{p}^{2}$ = 0.11], as well as a significant interaction between the three variables [*F*(1,43) = 14.96, *p* < 0.001, $\eta_{p}^{2}$ = 0.26]. When education years and SPM scores were used as covariances, significant interaction between the three variables was still observed [*F*(1,41) = 4.60, *p* = 0.038, $\eta_{p}^{2}$ = 0.10]. Further analysis demonstrated that the interaction of size of inducers and SF of stimuli was significant for both the younger [*F*(1,19) = 8.49, *p* = 0.009, $\eta_{p}^{2}$ = 0.31] and the older [*F*(1,24) = 6.67, *p* = 0.016, $\eta_{p}^{2}$ = 0.22] groups. Results of paired-samples *t* test showed that the illusion magnitude under each of the SF conditions was significant for both the younger [LSF: *t*(19) = 12.04, *p* < 0.001, *d* = 2.69; HSF: *t*(19) = 10.62, *p* < 0.001, *d* = 2.37] and the older [LSF: *t*(24) = 8.12, *p* < 0.001, *d* = 1.62; HSF: *t*(24) = 9.13, *p* < 0.001, *d* = 1.83] groups. For the younger group, the illusion magnitude was significantly larger for the LSF than for the HSF condition [*t*(19) = 2.91, *p* = 0.009, *d* = 0.65; see Figure 2B]. However, the older group showed the opposite results, that is, the illusion magnitude was significantly smaller for the LSF than for the HSF condition [*t*(24) = −2.58, *p* = 0.016, *d* = 0.52]. Moreover, the older group showed significantly reduced illusion strength than the younger group only for the LSF condition [*t*(43) = −2.89, *p* = 0.006, *d* = 0.87], but not for the HSF condition [*t*(43) = −0.43, *p* > 0.250, *d* = 0.13; see Figure 2B]. The disparity of illusion magnitudes between the LSF and the HSF conditions was significantly larger for the younger than for the older group [*t*(43) = 3.87, *p* < 0.001, *d* = 1.16; see Figure 2E].

**Table 2:** Comparisons of low- and high-spatial-frequency conditions with respect to accuracy (ACC) and the mean response times (RTs) for the gender discrimination task in Experiments 1 and 2.

			Younger			Older		
			*t*	*p*	*d*	*t*	*p*	*d*
Exp 1	Pre-test	ACC	−0.62	0.542	0.14	1.92	0.067	0.38
		RT	1.21	0.241	0.27	−0.04	0.966	0.01
	Test	ACC	−0.79	0.441	0.18	1.39	0.176	0.28
		RT	−0.17	0.867	0.04	−0.47	0.644	0.09
Exp 2	Pre-test	ACC	−1.79	0.089	0.40	1.40	0.174	0.28
		RT	0.19	0.850	0.04	0.46	0.653	0.09
	Test	ACC	0.96	0.347	0.22	−0.26	0.796	0.05
		RT	1.15	0.264	0.26	1.56	0.133	0.31

### Experiment 2

The proportions of correct responses on gender discrimination task were at chance level for both the younger [pre-test: *M* = 48.8%, *t*(19) = −1.33, *p* = 0.199, *d* = 0.30, BF_10_ = 0.39; test: *M* = 50.3%, *t*(19) = 0.37, *p* > 0.250, *d* = 0.08, BF_10_ = 0.18] and the older [pre-test: *M* = 50.8%, *t*(24) = 1.33, *p* = 0.195, *d* = 0.27, BF_10_ = 0.35; test: *M* = 50.3%, *t*(24) = 0.37, *p* > 0.250, *d* = 0.07, BF_10_ = 0.16; see Figure 2C] groups. The difference between the LSF and HSF conditions was not significant with accuracy and RTs during the pre-test and the test phases for the two age groups (Table 2). The disparity of RTs between the LSF and HSF conditions was comparable for the two age groups during both the pre-test [*t*(43) = −0.27, *p* > 0.250, *d* = 0.08, BF_10_ = 0.23] and the test [*t*(43) = −0.65, *p* > 0.250, *d* = 0.19, BF_10_ = 0.27] phases. For the size matching task, results from ANOVA demonstrated a significant main effect of size of inducers [*F*(1,43) = 170.00, *p* < 0.001, $\eta_{p}^{2}$ = 0.80], a non-significant main effect of SF of stimuli [*F*(1,43) = 0.35, *p* > 0.250, $\eta_{p}^{2}$ = 0.01], and a significant interaction between the three variables [*F*(1,43) = 24.01, *p* < 0.001, $\eta_{p}^{2}$ = 0.36]. When education years and SPM scores were used as covariances, significant interaction between the three variables was still observed [*F*(1,41) = 5.23, *p* = 0.027, $\eta_{p}^{2}$ = 0.11]. Further analysis demonstrated that the interaction of size of inducers and SF of stimuli were significant for both the younger [*F*(1,19) = 15.91, *p* = 0.001, $\eta_{p}^{2}$ = 0.46] and the older [*F*(1,24) = 9.31, *p* = 0.005, $\eta_{p}^{2}$ = 0.28] groups. Results of paired-samples *t* test showed that the illusion magnitude under each of the SF conditions was significant for both the younger [LSF: *t*(19) = 10.20, *p* < 0.001, *d* = 2.28; HSF: *t*(19) = 8.70, *p* < 0.001, *d* = 1.95] and the older [LSF: *t*(24) = 7.58, *p* < 0.001, *d* = 1.52; HSF: *t*(24) = 7.58, *p* < 0.001, *d* = 1.52] groups. For the younger group, the illusion magnitude was significantly larger for the LSF than for the HSF condition [*t*(19) = 3.99, *p* = 0.001, *d* = 0.89; see Figure 2D]. However, the older group showed the opposite results, i.e., the illusion magnitude was significantly smaller for the LSF than for the HSF condition [*t*(24) = −3.05, *p* = 0.005, *d* = 0.61]. Moreover, significant difference between the two age groups was observed only for the LSF condition [*t*(43) = 4.39, *p* < 0.001, *d* = 1.32], but not for the HSF condition [*t*(43) = 0.50, *p* > 0.250, *d* = 0.15; see Figure 2D]. The disparity of illusion magnitudes between the two SF conditions was significantly larger for the younger than for the older group [*t*(43) = 4.90, *p* < 0.001, *d* = 1.47; see Figure 2F]. Furthermore, the disparity of illusion magnitude between the LSF and HSF conditions in Experiment 2 was comparable to that in Experiment 1 for both the younger [*t*(19) = 0.88, *p* > 0.250, *d* = 0.20, BF_10_ = 0.25] and the older [*t*(24) = −0.76, *p* > 0.250, *d* = 0.15, BF_10_ = 0.20] groups.

## Discussion

By using low- and high-pass filtered images of faces and the Ebbinghaus illusion, the current study investigated age-related changes of the LSF bias in context sensitivity. The results showed that, for the younger participants, prior exposure to LSF faces increased the illusion strength relative to prior exposure to HSF ones. However, for the older participants, the opposite effect was observed, that is, prior exposure to LSF faces decreased the illusion strength relative to HSF ones. Moreover, a significant age group difference was observed only with prior exposure to LSF faces but not to HSF faces (Experiment 1). Similar patterns of results were observed when the filtered faces were rendered invisible with backward masking (Experiment 2), and the magnitudes of age-related decline were comparable to the visible condition.

Numerous studies support the right hemisphere hypothesis of cognitive aging, which claims that cognitive functions which correlate with the right hemisphere decline faster than those confined to the left hemisphere (Brown and Jaffe, 1975; Dolcos et al., 2002). A longitudinal magnetic resonance imaging study of older adults demonstrates that the right hemisphere shows greater overall gray matter loss compared to the left hemisphere, and the relative loss is most pronounced for the inferior frontal and anterior temporal regions (Resnick et al., 2003). Similarly, Jockwitz et al. (2017) found predominant gray matter atrophy in the right as compared to the left fronto-parietal regions which are encompassed in the default mode network. The age-related decline of functional connectivity between prefrontal and parietal regions also shows hemispheric asymmetry, with the connectivity decline being more pronounced in the right than the left hemisphere (Li et al., 2009). Meanwhile, converging psychophysical studies also suggest that the right hemisphere declines more than the left hemisphere with advancing age. For instance, it has been found that visuospatial cognition which is preferentially associated with the right hemisphere is generally more affected by aging than verbal cognition which is primarily associated with the left hemisphere (Lawrence et al., 1998; Jenkins et al., 2000). In a horizontal bisection line task, more bisection errors were observed for the older participants than for the younger ones when the flankers were presented in the left but not in the right hemispace (Chieffi et al., 2014). Moreover, Prodan et al. (2007) found that older adults demonstrated a markedly decreased ability to identify upper facial emotion of a blend presented to the left visual field, but were able to easily identify the lower facial emotion of a blend when presented to the right visual field, suggesting a relative right hemisphere aging effect. In line with the above evidence, our results showed that the age-related decline of context sensitivity was observed only with prior exposure to LSF but not to HSF information. The processing of SF information has been found to exhibit hemispheric specialization, with the processing of LSF and HSF information being predominantly associated with the right and left hemisphere, respectively (Kitterle et al., 1990; Christman et al., 1991; Peyrin et al., 2004; Kauffmann et al., 2014). Therefore, the current study extends the evidence in favor of the right hemi-aging model by showing that the effect of LSF bias on context sensitivity was reduced with increasing age.

A specialized subcortical visual pathway from the superior colliculus and the pulvinar to the amygdala has been postulated to detect threat-related signals outside awareness (Jiang and He, 2006; Hedger et al., 2015; Chen et al., 2016). The subcortical processing of threatening information has been suggested to be mainly carried out by LSF channels. Specifically, Vuilleumier et al. (2003) found that both the amygdala and the thalamic-collicular cluster showed significant response to LSF fearful faces, but not to HSF fearful faces. It has been found that older adults show intact mimicry response to invisible negative faces relative to younger adults (Bailey and Henry, 2009). Though normal aging does not markedly impair the structure and function of the amygdala (Soininen et al., 1994; Good et al., 2001; Grieve et al., 2005; St. Jacques et al., 2010), it does take effect on the functional connectivity of the amygdala with cortical regions. For instance, St. Jacques et al. (2010) found that older adults’ right amygdala showed comparative activity for negative stimuli relative to younger adults; meanwhile, reduced functional connectivity between the right amygdala and posterior regions including parahippocampus and visual cortex was observed for older adults in contrast to younger adults. Therefore, in the current study, significant age-related decline was only observed with prior exposure to LSF faces and was independent of the awareness of the LSF faces, suggesting that the magnocellular projections from subcortical areas (such as the amygdala) to cortical regions, such as the right occipitotemporal areas (Peyrin et al., 2004), might also be vulnerable to age-related changes.

In summary, our results demonstrate that the LSF-related enhancement of context sensitivity which was observed for the younger adults was significantly reduced for the older adults, and this age-related decline was independent of the awareness of LSF information. Our findings support the right hemi-aging model and suggest that both cortical regions in the right hemisphere and the magnocellular projections from subcortical to cortical regions might change with advancing age.

## Data Availability

The datasets generated for this study are available on request to the corresponding author.

## Ethics Statement

The studies involving human participants were reviewed and approved by the institutional review board of Liaoning Normal University. The patients/participants provided their written informed consent to participate in this study. Written informed consent was obtained from the individual(s) for the publication of any potentially identifiable images or data included in this article.

## Author Contributions

LC designed the study. AW and SZ collected the data. AW analyzed the data. LC, AW, and WL drafted the manuscript.

### Conflict of Interest Statement

The authors declare that the research was conducted in the absence of any commercial or financial relationships that could be construed as a potential conflict of interest.

## Footnotes

^1^For the global processing, the global forms of hierarchical stimuli are the targets. For the local processing, the local forms of hierarchical stimuli are the targets.

^2^Hierarchical stimuli consist of large letters/digits/geometric figures constructed from smaller letters/digits/geometric figures.
